# Advanced Composite of Large Cell Neuroendocrine Carcinoma and Squamous Cell Carcinoma: A Case Report of Uterine Cervical Cancer in a Virgin Woman

**DOI:** 10.1155/2013/921384

**Published:** 2013-08-24

**Authors:** Ryusuke Murakami, Iemasa Kou, Kenjiro Date, Hirofumi Nakayama

**Affiliations:** ^1^Department of Obstetrics and Gynecology, Hiroshima General Hospital of West Japan Railway Company, 3-1-36 Futabanosato, Higashiku, Hiroshima 732-0057, Japan; ^2^Department of Pathology, Hiroshima General Hospital of West Japan Railway Company, 3-1-36 Futabanosato, Higashiku, Hiroshima 732-0057, Japan

## Abstract

Large cell neuroendocrine carcinoma (LCNEC) of the uterine cervix is very rare and aggressive. The prognosis is very poor despite multimodal treatment. We report a virgin woman with FIGO stage 4b LCNEC of uterine cervix coexisting with squamous cell carcinoma. An early thirties virgin woman presented with 2-month history of abdominal pain. A chest X-ray showed multiple lung metastatic tumors. A vaginal smear showed malignant cells, and a biopsy specimen had features of LCNEC. The tumor showed trabecular patterns. Tumor cells possessed a moderate amount of cytoplasm, prominent nucleoli, and large nuclei. The tumor cells are stained positive for synaptophysin, chromogranin A, and neuron specific enolase (NSE). The invasive tumor cells in connection with cervical squamous epithelium were focally positive for 34bE12. We made a diagnosis of composite LCNEC and nonkeratinizing squamous cell carcinoma. High-risk HPV test was negative with hybridized captured method 2.

## 1. Introduction

Large cell neuroendocrine carcinoma (LCNEC) is a subtype of neuroendocrine tumors, along with small cell neuroendocrine carcinoma (SCNEC) and carcinoid tumors. LCNEC of the uterine cervix is very rare, highly aggressive, and usually results in unfavorable outcomes. 

We describe a virgin woman with cervical tumor whose vaginal pap smear had atypical cells probably malignant, and the biopsy specimen revealed a diagnosis of composite LCNEC and squamous cell carcinoma. 

An LCNEC of uterine cervix coexisting with squamous cell carcinoma was encountered in a virgin woman, very rare case to be reported in the world.

## 2. Case Report

An early 30s virgin woman presented with 2-month history of upper and lower abdominal pain and 1-month history of right inguinal and leg pain without any genital irregular bleeding. She had already had two medical consultations in her local clinics and received an abdominal ultrasonographic examination and a pelvic magnetic resonance imaging (MRI), which showed no remarkable findings. She received a chest X-ray in another clinic, which showed multiple bilateral lung nodules. Then she received a chest and abdominal computed tomography (CT) scan 2 weeks prior to our consultation, which showed multiple tumors in bilateral lungs, para-aortic lymph nodes and Virchow lymph nodes swelling, pelvic mass behind the uterus, and slight ascites. 

**Figure 1:**
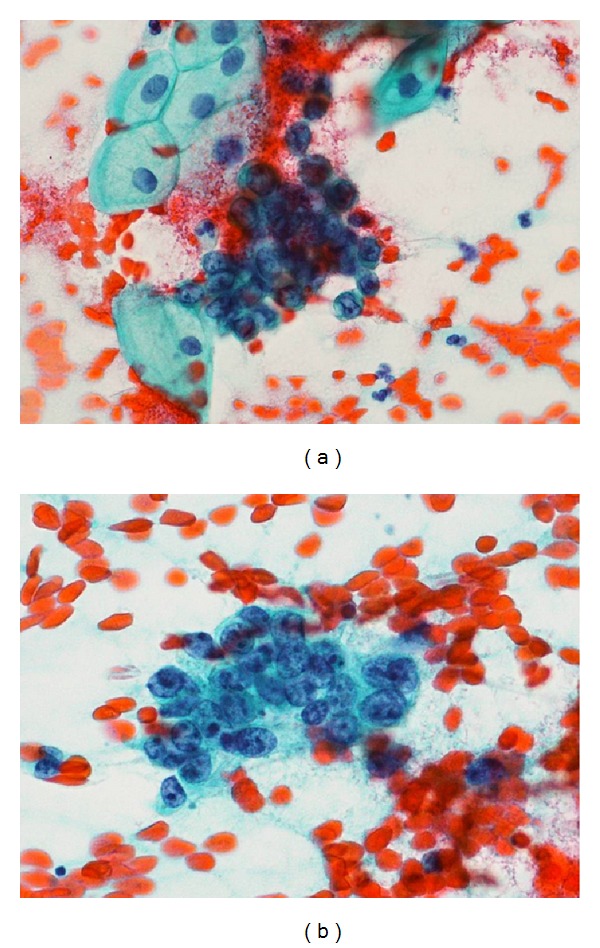
Cytologic features of LCNEC. (a) Pap smear of the vagina shows atypical oval cells with scant cytoplasm and hyperchromatic coarse nuclei. Surface squamous cells showed enlarged nuclei with fine granular chromatin. (b) Pap smear of the uterine cervical tumor shows tumor cells in overlapping clusters containing oval nuclei with coarsely clumped chromatin, irregular-shaped nucleoli, and moderate granular cytoplasm.

**Figure 2:**
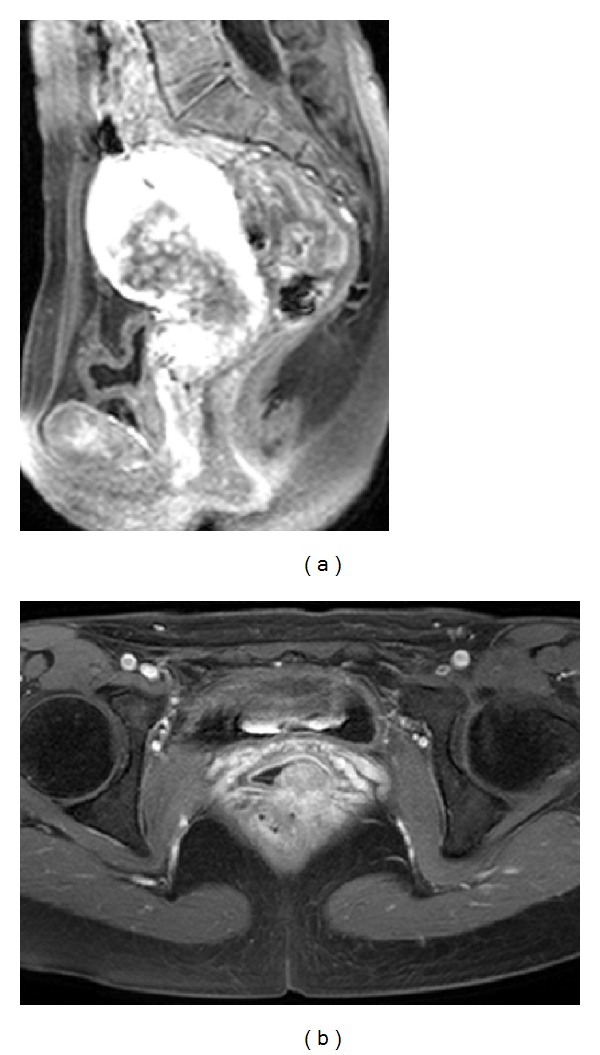
Pelvic MRI. (a) Sagittal fat-suppressed T1-weighted MR image after gadolinium administration showed region heterogeneously poor enhanced in cervix, vagina, and uterine corpus myometrium which were irregularly enhanced in late phase. (b) Axial fat-suppressed T1-weighted MR image after gadolinium administration showed cervical mass homogeneously enhanced in the posterior wall from the uterine cervix to the vagina.

She came to our gynecological department with no previous coital experience. A speculum transvaginal examination was not able to be performed, and then a vaginal exfoliative cytology specimen had atypical cells probably malignant (Figure 1). A pelvic MRI contrast revealed an invasive tumor in the cervix, vagina, and uterine corpus myometrium which were irregularly enhanced in late phase (Figure 2). The MRI revealed bilateral ovarian, multiple pelvic bone, and pelvic lymph node metastasis. A positron emission tomography (PET) scanning showed glucose metabolism strongly concentrated in uterine cervix, endometrium, widespread pelvic, para-aortic, pulmonary and Virchow lymph nodes, pelvic bone, and bilateral lungs. Serum tumor markers were elevated: CA125 199.9 mU/mL, CA19-9 172 U/mL, CYFRA 32 ng/mL, SCC 1.6 ng/mL, and NSE 93.7 ng/mL. 

**Figure 3:**
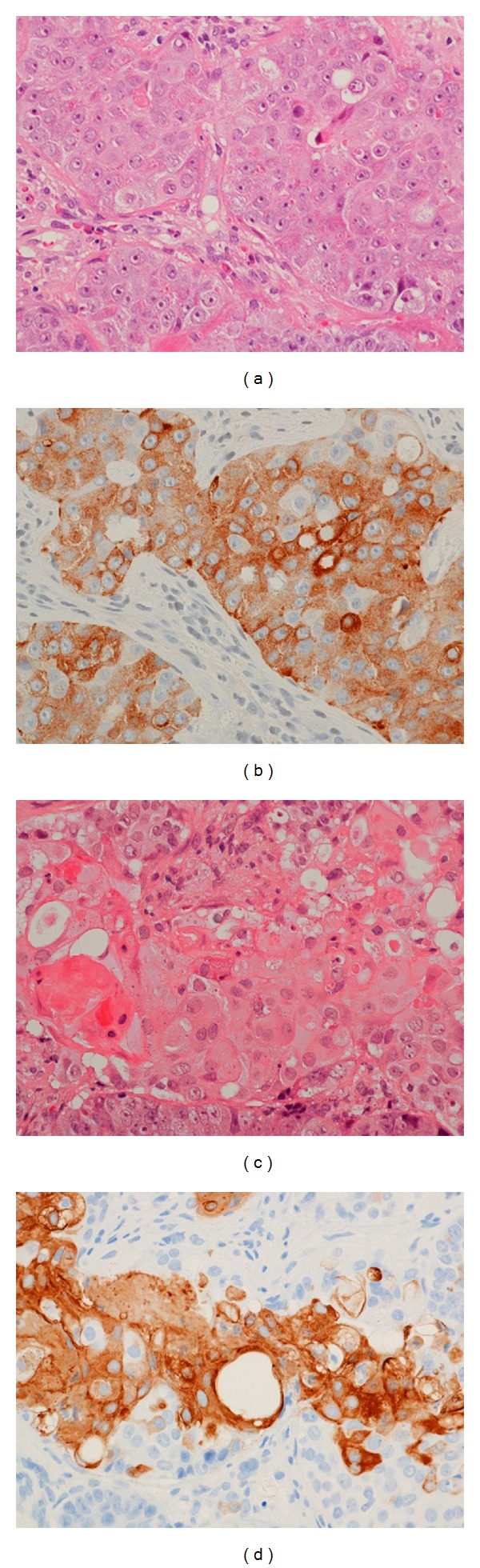
Histology of LCNEC with squamous cell carcinoma. (a) HE stain shows that tumor cells with trabecular patterns possess prominent nucleoi and large nuclei and a moderate amount of cytoplasm with eosinophil granule. (b) Synaptophysin is positive in the LCNEC. (c) HE stain shows squamous cancer cells without any keratin pearl. (d) The invasive tumor nests have 34bE12 positive neoplastic squamous cells.

A wedge biopsy taken from the uterine cervix under intravenous anesthesia disclosed a large cell neuroendocrine carcinoma (LCNEC) and squamous cell carcinoma. The tumor cells with trabecular patterns possessed a moderate amount of cytoplasm with prominent nucleoli and large nuclei. Immunohistochemistry revealed diffuse staining of the LCNEC component against antibodies stained positive for synaptophysin, chromogranin A, and neuron specific enolase (NSE), and partial nuclear staining for p16, p63, and TTF-1. The mitotic activity was more than 3 mitotic figures per 10 high-power fields. The 34bE12 positive tumor cells were scattered (Figure 3).

The diagnosis of composite LCNEC and nonkeratinizing squamous cell carcinoma was made. The FIGO stage 4b uterine cervical cancer was diagnosed. High-risk human papillomavirus (HPV) test was negative with hybridized captured method 2. 

The initial chemotherapy regimen consisted of irinotecan (60 mg/m^2^ on days 1, 8) and cisplatin (60 mg/m^2^ on day 1) every 3 weeks. The treatment started 2 weeks after our consultation. The foci of lung metastasis decreased in number and size remarkably with chest X-rays. The PET-CT showed regression in lung tumors and Virchow lymph nodes but progression of disease especially in pelvic bone metastasis, liver metastasis, and multiple brain metastasis. The patient died 4 months and 2 weeks after diagnosis in spite of multimodal treatments.

## 3. Discussion

According to the classification adopted by the College of American Pathologists and the National Cancer Institute in 1997, cervical neuroendocrine tumors are categorized into four distinctive subtypes: SCNEC, LCNEC, typical, and atypical carcinoid tumors [1]. This terminology, which is identical to that used for the classification of pulmonary neuroendocrine tumors, has been incorporated into the latest World Health Organization Classification of tumors of the Female Genital Tract. LCNEC is characterized by cells of large size, polygonal shape and low nuclear-cytoplasmic ratio, mitotic activity in excess of 10 mitotic figures per 10 high-power fields with areas of necrosis, and immunohistochemical or ultrastructural evidence of neuroendocrine differentiation [2].

The cytological features of LCNEC were reported in which tumor cells in overlapping clusters have oval to rounded nuclei with fine granular to coarsely clumped chromatin, two or more irregular-shaped nucleoli, and moderate to abundant cytoplasm. The size of nucleus is the largest among the neuroendocrine tumors, 3–5 times larger than the diameters of small lymphocytes [3, 4]. 

LCNEC is very rare, and the incidence has been reported to be 0.087%–0.6% of all primary cervical malignancy in the literature [5, 6]. Embry et al. reviewed 62 patients with LCNEC; median age was 37 (range 21–75) [7]. FIGO stage was as follows: 58% had stage 1 disease, 16% had stage 2 disease, 2% had stage 3 disease, and 8% had stage 4 disease. Of all patients, 58% died of the disease, 26% had no evidence of the disease, and 3% were alive with disease. The overall median survival was 16.5 months (0.5–151 months). 

Median overall survival for stages 1, 2, 3, and 4 cancers was 19, 17, 3, and 1.5 months, respectively [7]. Most presented with early stage disease and received multimodal treatment, yet the outcome was poor with early metastasis. Thus recognition and accurate diagnosis of this rare tumor are essential for formulating an effective treatment plan. The correct interpretation depends on a high index of suspicion and appreciation of the features of neuroendocrine differentiation in the nonsquamous or adenomatous component of the tumor. These features include uniform medium-to-large cells in a trabecular of insular pattern, eosinophilic granules in the cytoplasm, and immunohistochemical staining for neuroendocrine markers [8].

The surgical treatment and chemotherapeutic regimens for cervical LCNEC seemed to follow the schematic outline of cervical SCNEC. However, limited experiences preclude a definite conclusion regarding the optimal chemotherapeutic option. For cervical LCNEC, treatment for early stage diseases is radical surgery. Chemotherapy with or without radiation is additionally used in early stage disease or in advanced stage diseases [7–9]. We selected the 1st line chemotherapy for cervical LCNEC following the regimens for LCNEC of the lungs. The common chemotherapy was platinum-based combinations of etoposide or irinotecan [10].

TTF-1 is a nuclear transcription factor which is expressed in thyroid and lung epithelial cells and is commonly used as a marker of thyroid carcinoma, pulmonary adenocarcinoma, and pulmonary neuroendocrine neoplasms. Neuroendocrine tumors arising in other organs are also positive for TTF-1. TTF-1 positivity could potentially have been misinterpreted as evidence of a pulmonary origin for the tumors with metastasis to the cervix, especially in the case with multiple lung lesions [11].

Most LCNEC patients had HPV-16 or -18 infection [6]. It is difficult to show HPV negativity because of the limitation of the biopsy specimens in this case. Partial positivity for p16 can be associated with high-risk HPV related region. However, high-risk HPV test was negative with hybridized captured method 2.

P63 staining is regarded as a useful immunohistochemical marker of squamous differentiation within a cervical carcinoma. In this case LCNEC component was partially positive for p63. P63 nuclear immunoreactivity may not be taken as unequivocal evidence of a squamous carcinoma. The squamous cell component was focally positive for 34bE12, and LCNEC component was diffusely negative for 34bE12. Then this result may be explained that 34bE12 is a more specific marker for squamous cell component compared with p63 in LCNEC.

LCNEC of uterine cervix coexisting with squamous cell carcinoma was encountered even in a virgin woman. It is important to establish a diagnosis of LCNEC on cytology and small biopsy specimens and resections due to the aggressive nature of the neoplasm.
